# siRNA delivery targeting to the lung *via* agglutination-induced accumulation and clearance of cationic tetraamino fullerene

**DOI:** 10.1038/srep04916

**Published:** 2014-05-12

**Authors:** Kosuke MINAMI, Koji OKAMOTO, Kent DOI, Koji HARANO, Eisei NOIRI, Eiichi NAKAMURA

**Affiliations:** 1 Department of Chemistry, The University of Tokyo, 7-3-1 Hongo, Bunkyo-ku, Tokyo 113-0033, Japan; 2 Department of Hemodialysis and Apheresis, University Hospital, The University of Tokyo, 7-3-1 Hongo, Bunkyo-ku, Tokyo 113-8655, Japan; 3 Present address: International Center for Materials Nanoarchitectonics, National Institute for Materials Science, 1-1 Namiki, Tsukuba 305-0044 Japan.

## Abstract

The efficient treatment of lung diseases requires lung-selective delivery of agents to the lung. However, lung-selective delivery is difficult because the accumulation of micrometer-sized carriers in the lung often induces inflammation and embolization-related toxicity. Here we demonstrate a lung-selective delivery system of small interfering RNA (siRNA) by controlling the size of carrier vehicle in blood vessels. The carrier is made of tetra(piperazino)fullerene epoxide (TPFE), a water-soluble cationic tetraamino fullerene. TPFE and siRNA form sub-micrometer-sized complexes in buffered solution and these complexes agglutinate further with plasma proteins in the bloodstream to form micrometer-sized particles. The agglutinate rapidly clogs the lung capillaries, releases the siRNA into lung cells to silence expression of target genes, and is then cleared rapidly from the lung after siRNA delivery. We applied our delivery system to an animal model of sepsis, indicating the potential of TPFE-based siRNA delivery for clinical applications.

Although tissue-specific drug delivery attracts much attention in current therapeutics^1^,^2^, there has been limited success in methods for lung-selective drug delivery. Pulmonary inhalation, which delivers the drug to the alveolar surface, has been widely investigated, however, is generally inefficient pathway for delivering the drugs, especially the large or hydrophilic drugs due to the lung mucus barrier^3^, and often induces lung fibrosis^4^,^5^. Because they are the narrowest capillaries in the body, alveolar capillaries serve as target sites of another strategy aimed at accumulation of drug-carrying microspheres to be taken up by alveolar macrophages. Liposomes made of cationic lipids have been used as the carrier for lung-targeting drug delivery *in vivo*, but there are problems such as significant dose-dependent toxicity, especially pulmonary inflammation^6^, and clogging of capillaries, which causes chronic obstructive pulmonary emphysema^7^ and arterial embolism^8^,^9^.

Microspheres need to be larger than ca. 6 μm to be accumulated in the lung capillaries and to be cleared after the drug delivery to avoid embolization-related toxicity^10^,^11^. On the other hand, the drug-carrier conjugates need to be in 100 nm to 1 μm size for efficient uptake by lung cells^12^,^13^. To solve the dilemma on the optimal sizes for lung-targeted delivery, stable submicrometer-sized complex prepared prior to intravenous injection must grow into micrometer-sized particles under physiological conditions, release drugs at lung capillaries, and escape from the lung when the drug delivery is complete. In light of the diverse aggregation behavior of fullerenes in water^14^, their ability to protect DNA from degradation, and *in vitro* and *in vivo* gene delivery^15^,^16^,^17^, we hypothesized that a cationic fullerene could serve as a carrier for the delivery of small interfering RNA (siRNA) for RNA interference (RNAi) therapeutics *in vivo*^18^,^19^. We report here on the lung-specific delivery of siRNA using a cationic fullerene, tetra(piperazino)fullerene epoxide (TPFE) (Fig. 1)^20^. This delivery agent agglutinated with siRNA and plasma proteins in the bloodstream to form >6-μm particles, which accumulated in the narrow lung capillaries. The siRNA was then released into lung cells, where it induced gene silencing, and the TPFE was cleared quickly from the lung. We could confirm the time course of knockdown by histological analysis because of the unique brownish-yellow color of TPFE.

This new delivery protocol has considerable therapeutic potential as shown in our model of sepsis-induced acute lung injury induced by intratracheal lipopolysaccharide (LPS) injection in C3H/HeN (*TLR4^+/+^*) and C3H/HeJ (*TLR4^–/–^*) mice. RNAi therapeutics has gained significant attention and has been translated into clinical trials^21^. However, siRNA is unstable in serum and its delivery needs effective carrier vectors that compact and protect RNA^22^. With its ability to protect RNA, organ selectivity, and low toxicity, TPFE will serve as a useful alternative to the current cationic lipid vectors^23^.

## Results

### Agglutination of TPFE–siRNA complex in the physiological environment

We first evaluated the size of TPFE–siRNA complexes in buffer and in serum. siRNA-targeting enhanced green fluorescent protein (EGFP) was used. We mixed an aqueous solution of TPFE, which is brown in color because of fullerene's visible light absorption, and siRNA in a buffer with a TPFE-to-base pair ratio (*R*) of 20. The size of the TPFE–siRNA complex was determined by dynamic light scattering (DLS) analysis (Fig. 2). The average size of the complex was 160 nm, which is a suitable size for cellular uptake. The TPFE–siRNA complex did not change in size in the aqueous buffer for at least 6 h (white bars in Fig. 2). By contrast, the average size of the TPFE–siRNA complex increased rapidly to >6 μm after coincubation with mouse serum (black bars in Fig. 2). This was probably caused by the electrostatic interaction between the cationic TPFE–siRNA complex and anionic plasma proteins. This brownish emulsion did not show any signs of precipitation for at least 6 h.

### siRNA delivery efficiency of TPFE–siRNA complexes *in vivo*

The *in vivo* biodistribution of injected siRNA was evaluated by the knockdown of EGFP mRNA expression as measured by quantitative real-time reverse transcription polymerase chain reaction (RT-PCR). TPFE–siRNA complexes were injected intravenously into the EGFP-overexpressing mice. EGFP-targeted siRNA (siGFP) and scrambled siRNA (siNEG) were used. At 24 h after the injection, total RNA was extracted from the brain, heart, lung, liver, kidney, and spleen. No significant knockdown was observed in any tissue except the lung, which showed a significant 62% knockdown (Fig. 3). Together with the DLS results (Fig. 2), this result suggests that the TPFE–siRNA complexes agglutinated in the bloodstream, accumulated in the lung, and delivered the siRNA into the lung tissue.

The clogging of the lung capillaries by TPFE–siRNA complexes was observed visually by analyzing the lung histology. TPFE appeared as a brownish-orange-colored region in the paraffin-embedded lung sections (Fig. 4, see also Fig. 5a–e). Histology showed that TPFE–siRNA was distributed first in the lung capillaries soon after the injection and was then internalized rapidly into the lung cells within 3 h (Fig. 4b,f). The clogged material and the TPFE color in the lung capillaries disappeared 12 h after the injection (Fig. 4c,g).

We studied the relationship between the accumulation of TPFE and the RNAi efficacy in the lung, which indicated quick release of siRNA from the TPFE–siRNA complex. The colored TPFE allowed us to evaluate quantitatively the time course of TPFE accumulation and clearance without recourse to staining (Fig. 5a–e). The accumulation of TPFE increased rapidly to ca. 400 μm^2^ per high-power field (100 × 100 μm^2^) and then decreased to <50 μm^2^ at 24 h (Fig. 5f). The time-dependent knockdown efficiency was examined using quantitative real-time RT-PCR by comparing the EGFP mRNA expression levels between siGFP- and siNEG-injected mice. The knockdown efficiency in the lung increased after injection of TPFE–siRNA and was maximum at 24 h (Fig. 5g, black bars), whereas no time dependence was found in the siNEG-injected group (Fig. 5g, white bars). In our previous investigations, TPFE produced no acute toxicity in vitro or *in vivo*^16^,^20^. Thus, the complex was injected intravenously into mice (*N* = 8). In this study, the TPFE–siRNA complex was found to be nontoxic: no acute organ toxicity or death was observed for 2 weeks after the injection.

### Delivery of TLR4-targeted siRNA for therapeutic application

We evaluated the application of mouse toll-like receptor 4 (TLR4)-targeted siRNA delivery by TPFE to the suppression of neutrophil accumulation induced by LPS injection in the lung. siRNA targeting TLR4 (siTLR4) was injected into wild-type mice (C3H/HeN, *TLR4^+/+^*) and into mice harboring the *TLR4* missense mutation (C3H/HeJ, *TLR4^–/–^*) as the negative control. We also carried out a control experiment using siGFP as non-targeting siRNA. LPS was injected intratracheally 24 h after intravenous injection of the TPFE–siTLR4 complex, and lung specimens were collected 6 h after the LPS injection. The accumulation of neutrophils was evaluated by lung pathology analysis and by measuring myeloperoxidase (MPO) activity (Fig. 6a–e). Both neutrophil sequestration and MPO activity in the lung of siTLR4-injected mice were significantly lower than the siGFP-injected mice, and were as low as the mice harboring the *TLR4* missense mutation, C3H/HeJ (*TLR4^–/–^*). The expression of TLR4 mRNA was determined by real-time RT-PCR (Fig. 6f), which showed that the TLR4 mRNA was knocked down by siTLR4 in the lung. These results indicate that siTLR4 delivered by TPFE knocked down TLR4 and significantly suppressed the accumulation of the neutrophils by the LPS-TLR4 signaling pathway.

## Discussion

Since its discovery^18^, RNAi has emerged as one of the most powerful tools for the sequence-specific suppression of genes using siRNA. siRNA is a 21–23-nucleotide non-coding RNA, which has attracted attention as an alternative method to traditional gene therapy using DNA. Some RNAi therapeutics have been translated into clinical trials^21^. Unlike DNA, which is relatively stable in the physiological environment, RNAs, including siRNA, are degraded rapidly by serum nucleases and hydrolysis. The efficient delivery of siRNA *in vivo* requires effective carriers that compact and protect RNA^22^. Our previous finding that TPFE can protect and deliver 1–4 kbp of DNA *in vivo*^16^ prompted us to examine a more challenging target—the *in vivo* delivery of siRNA, which is more vulnerable to degradation.

Because of its tetracationic property, TPFE aggregates with DNA molecules with a TPFE-to-base pair ratio (*R*) of ca. 5 to form submicrometer-sized globules/particles, in which the TPFE molecules act like histone molecules to fold the long DNA chain into a globule^24^. TPFE has much lower toxicity than the cationic lipid equivalents. The higher hydrophobicity of fullerenes compared with alkyl chains in lipids enables TPFE to form a stable TPFE–DNA complex with a size of 50 nm, in which TPFE provides much better protection of the DNA against nucleases than do cationic lipids^25^. Upon complexation of TPFE with 1–6-kbp DNA at *R* = 5 in serum, TPFE–DNA globules interacted with plasma proteins but retained their 100-nm size for hours in the bloodstream. The delivery occurred predominantly through the reticuloendothelial system including the liver and spleen^16^.

**Figure 1:**
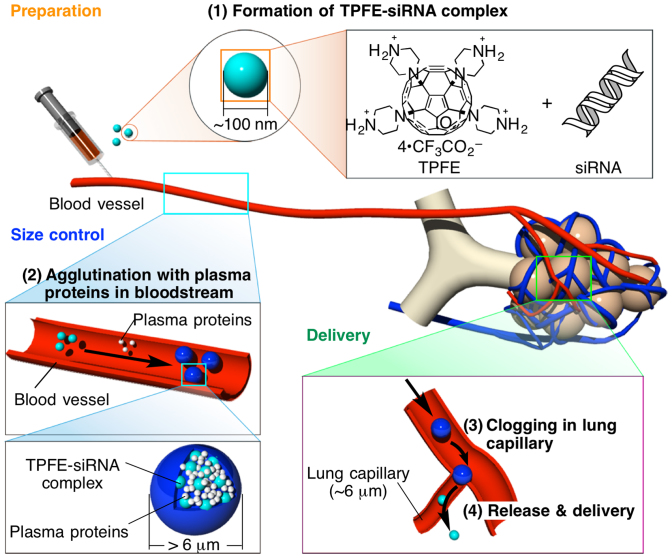
The rationale for lung-specific delivery of siRNA mediated by tetra(piperazino)fullerene TPFE. (1) TPFE aggregates with siRNA to form ca. 100 nm-sized TPFE–siRNA complexes (light blue particles) by electrostatic interaction. (2) The complexes agglutinate with plasma proteins (white particles) in the bloodstream to form >6-μm particles (blue particles). (3) The particles clog and accumulate in the narrow lung capillaries. (4) The TPFE–siRNA complexes were delivered into lung cells, and the siRNA was released into the lung cells. Light-blue, white, and blue particles indicate TPFE–siRNA complexes, plasma proteins, and agglutinated particles, respectively.

**Figure 2:**
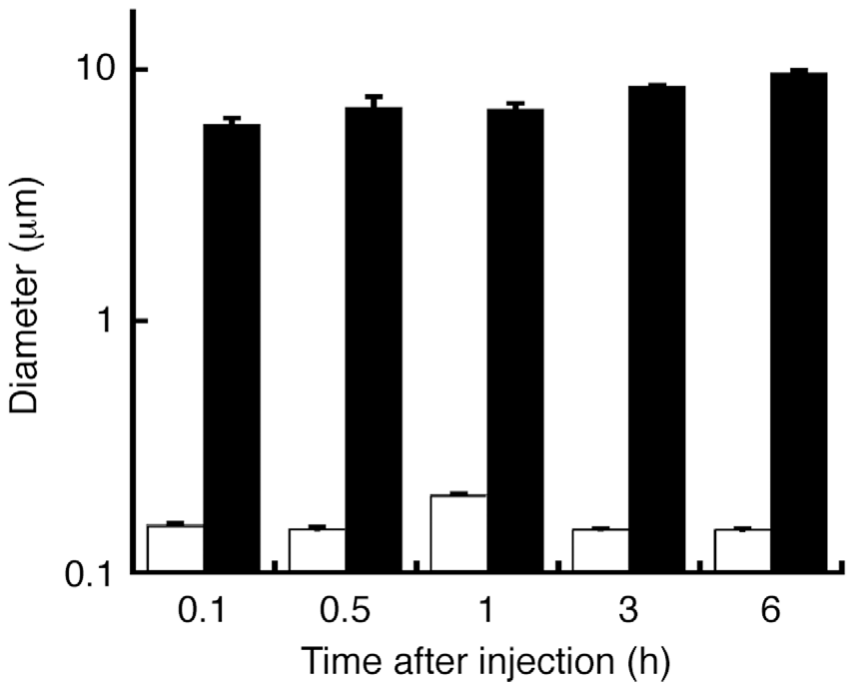
Time-dependent DLS analysis of TPFE–siRNA. The change in size was monitored after injection of the TPFE–siRNA complex into PBS buffer without mouse serum (white) or into PBS buffer containing mouse serum (black). The TPFE–siRNA complex agglutinated in the serum. Error bars indicate ±SD (*N* = 3 for each group).

**Figure 3:**
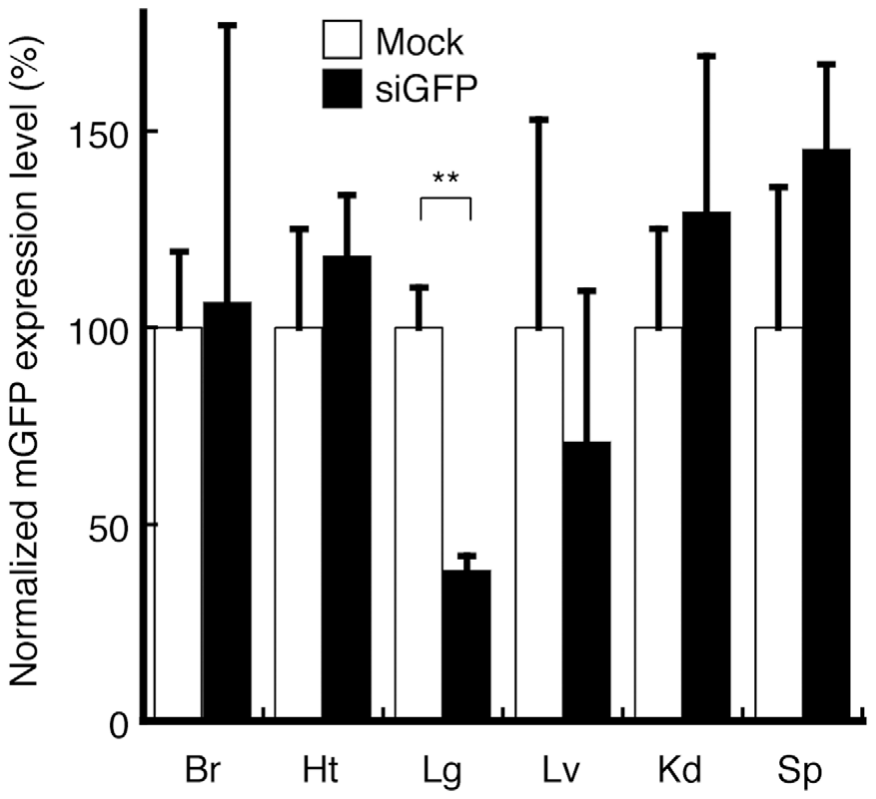
*In vivo* biodistribution of injected siRNA in each organ. At 24 h after the injection of TPFE–siRNA complexes, knockdown of mRNA expression of EGFP was detected by real-time RT-PCR. (*N* = 4 for each group). Black and white bars indicate the siGFP and siNEG as a negative control, respectively. Error bars are SEM. ** *P* < 0.005 versus siNEG-injected group. Abbreviations: Br, brain; Ht, heart; Lg, lung; Lv, liver; Kd, kidney; Sp, spleen.

**Figure 4:**
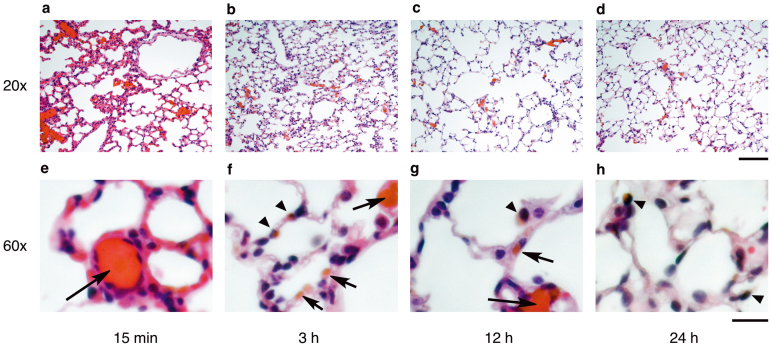
*In vivo* biodistribution of TPFE–siRNA complexes in lung capillaries and cells. (a–h*)* Histological appearance of TPFE in mouse lungs sacrificed at 15 min (a), (e) and 3 (b), (f), 12 (c), (g), and 24 h (d), (h) after TPFE–siRNA injection. Hematoxylin and eosin were used as a counterstain. Brownish-orange-colored areas indicate TPFE. Arrows and arrowheads indicate localization of TPFE in the lung capillaries and cells, respectively. Original magnification used and scale bars: (a–d), 20× and 100 μm; (e–h), 60× and 20 μm.

**Figure 5:**
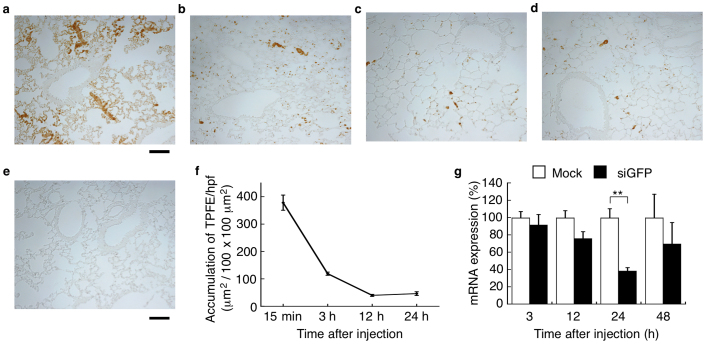
Time course of accumulation of TPFE and RNAi activity in mouse lung. (a–d) Histological appearance of TPFE in mouse lungs sacrificed at 15 min (a), 3 h (b), 12 h (c), and 24 h (d) after injection of TPFE–siRNA complexes. Brownish-orange colored area indicates the color of TPFE. Scale bars are 100 μm. (e) Histological appearance of unstained lung sections at 3 h after injection of saline. Scale bar is 100 μm. (f) Time-dependent TPFE accumulation determined by histology (*N* = 3). (g) RNAi activities of siGFP and scrambled siRNA are shown in black and white bars, respectively (*N* = 4 for 3, 12 and 24 h, *N* = 6 for 48 h). Error bars are SEM. ** *P* < 0.005 versus scrambled siRNA-injected group.

**Figure 6:**
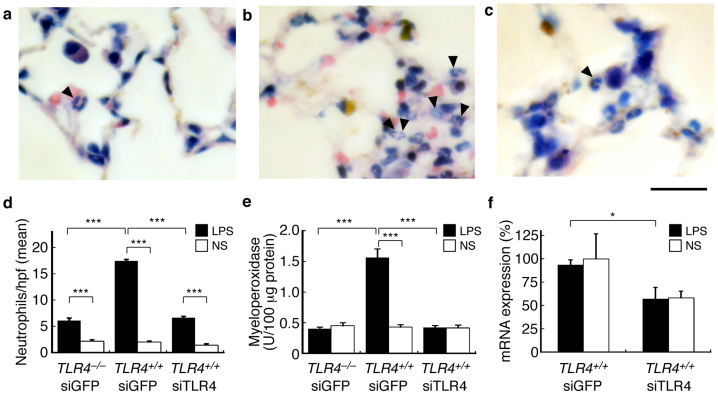
Suppression of neutrophil sequestration in the lung by lung-specific delivery of siTLR4 by TPFE. (a–c) Neutrophils stained with Giemsa stain. TPFE–siGFP-treated C3H/HeJ mice (TLR4*^–/–^*, a) and C3H/HeN mice (TLR4^+/+^, b), and TPFE–siTLR4-treated C3H/HeN mice (TLR4^+/+^, c) treated by intratracheal LPS injection are shown. Scale bar is 20 μm. (d–e) Quantitative analysis of neutrophil sequestration into the lung and lung MPO activity, respectively (*N* = 4 for each group). Black and white bars show the intratracheal treatment of LPS and normal saline, respectively. (f) Knockdown of mRNA expression of TLR4 was detected by a real-time RT-PCR analysis (*N* = 4 for each group). All error bars are SEM. * *P* < 0.05. *** *P* < 0.001.

Because of the very small size of siRNA (21–23 nucleotides), we envisioned a different scenario for RNA delivery. TPFE complexation with siRNA with *R* = 5 was precipitated completely, whereas complexes with *R* = 20, which had a particle diameter of 160 nm, were not precipitated and remained stable for 6 h in buffered solution. With *R* = 20, the particle comprised mainly TPFE and was thus cationic, and the siRNA would have acted as a glue connecting the TPFE aggregates to form a globule (see Fig. 1). In the bloodstream, the highly cationic TPFE–siRNA particles should form larger aggregates upon agglutination with plasma proteins than those formed from the TPFE–DNA complex, and hence should accumulate in the narrow lung capillaries (ca. 6 μm)^10^,^11^. We also hypothesized that the accumulated particles would disassemble when the glue, the submicrometer-sized particles composed of siRNA and TPFE, was removed by uptake into the lung cells.

The *in vivo* study of the biodistribution of the injected TPFE–siRNA complexes showed rapid accumulation of TPFE–siRNA complexes in the lung tissue and internalization into the cells within 3 h. A similar observation was reported by Shim et al. for the delivery of polymer–anticancer drug microspheres^9^. However, the most remarkable feature of the TPFE–siRNA complex is that it was cleared within 1 day, during which time the efficient knockdown of mRNA expression levels occurred in the lung with the maximum effect at 24 h after injection. Importantly, we could monitor the clearance of the drug carrier from the lung by taking advantage of the inherent brown color of TPFE, which could be distinguished in the unstained lung sections. The time course of the knockdown suggests that the siRNA started to function immediately after accumulation of TPFE–siRNA complexes in the pulmonary capillaries. These results are consistent with the prediction that the siRNA would be released rapidly from TPFE–siRNA aggregates into the lung cells through enzymatic acylation (neutralization) of the secondary amine groups in TPFE^20^. The released siRNA then bound to the RNA-induced silencing complex and cleaved the target mRNA, and the remaining TPFE was cleared from the lung cells and tissues. Although the eventual fate of the TPFE is unclear at this time, according to the pharmacokinetic studies of water-soluble fullerenes^26^,^27^, majority must have been excreted into the urine and feces.

Cationic lipids are used widely in drug and gene delivery protocols, but are not suitable for delivery to the lung. The lung capillary is the primary site for accumulation of micrometer-sized particles introduced by intravenous injection. Cationic liposomes injected into the bloodstream interact and aggregate with erythrocytes through both membrane fusion and electrostatic interaction, and accumulate immediately in the lung before distribution to other tissues^28^. This accumulation often causes toxic effects such as chronic obstructive pulmonary emphysema and arterial embolism. Fullerene is both hydrophobic and lipophobic^29^, and thus the TPFE aggregates do not fuse with the lipophilic erythrocyte membrane but prefer to aggregate with plasma proteins. As a result, spontaneous clearance from the lung capillaries via dissociation of the TPFE–siRNA complexes must have taken place. Rojanasakul et al. suggested that accumulation of the cationic liposomes causes increasing toxicity and inflammatory activity, which are mediated by reactive oxygen species (ROS) generated through the oxidative burst^6^. Notably, fullerene and its derivatives are potent scavengers of ROS^30^,^31^,^32^, and this unusual ability of fullerenes could be the reason for the lack of acute toxicity in TPFE-mediated RNA and DNA delivery. We therefore conclude that TPFE acts as both a delivery agent of siRNA to the lung via agglutination with plasma proteins and as a scavenger of ROS, thereby preventing pulmonary inflammation. Evaluation of the long-term toxicity is the next step toward application of fullerene-mediated siRNA delivery in humans.

The TLR4-targeted siRNA delivery by TPFE suppressed neutrophil accumulation in the lung induced by LPS treatment. Rapid accumulation of neutrophils in the lung, especially in the narrow lumen of the lung capillaries, plays a crucial role in acute lung injury caused by sepsis^33^. This pathological condition is mimicked by intratracheal LPS injection in animal models. LPS-induced activation of mammalian cells occurs through TLR4, the dominant LPS receptor. LPS induces endothelial injury, and neutrophils are sequestered in the lungs. Thus, in the wild-type mice C3H/HeN (*TLR4^+/+^*), LPS injection markedly increased neutrophil infiltration into the lung capillaries. By contrast, the mice harboring the *TLR4* missense mutation C3H/HeJ (*TLR4^–/–^*), whose macrophages are nonresponsive to LPS, showed no accumulation of neutrophils in the lungs after systemic LPS injection. Use of the TPFE–siRNA delivery system in the mouse model suppressed neutrophil accumulation and MPO activity in the lung to a level (0.42 U/100 μg lung protein) similar to that in the *TLR4*-mutated mice. Further evaluation is needed to clarify the potential applications of TPFE for siRNA delivery in animal models of diseases. Our results of therapeutic effects show that fullerene-based siRNA delivery has potential in clinical applications.

In summary, we have demonstrated that TPFE selectively delivers siRNA to the lung. The most notable observation was the accumulation of micrometer-sized particles of TPFE–siRNA–plasma protein in the lung capillaries and the clearance of TPFE by the size decrease of carrier vehicles after the siRNA was delivered to the lung tissue. The self-assembled scaffold of the three-component complex enables the dissociation into smaller particles suitable for the cellular uptake as well as the clearance of TPFE, which could be monitored histologically because of the unique brown color. Our results offer the strategy of lung-specific delivery, in which siRNA carriers agglutinate with plasma proteins in the bloodstream, accumulate in the lung capillaries, and release the siRNA to the lung cells for the treatment of sepsis and other lung diseases. The therapeutic potential of the new protocol was demonstrated through the successful delivery of TLR4-targeted siRNA (siTLR4) by the TPFE system and the suppression of neutrophil accumulation in the lung, indicating the clinical applicability of the fullerene-based siRNA delivery systems.

## Methods

### Preparation of TPFE–siRNA complexes

TPFE was synthesized by following the procedure reported previously^20^. siGFP (sense strand, 5′-CGGCAAGCUGACCCUGAAGUU-3′ and antisense strand, 5′-UGAACUUCUGGGUCUGCUUGC-3′); siTLR4 (sense strand, 5′-GUUCCAUUGCUUGGCGAAUGUUU-3′ and antisense strand, 5′-ACAUUCGCCAAGCAAUGGAACUU-3′); and siNEG (sense strand, 5′-CGGCAAGUCUCCCAAGGAGUU-3′ and antisense strand, 5′-UGAACUCCUUGGGAGACUUGC-3′) were purchased from Hokkaido System Science Co., Ltd. TPFE dissolved in 2 mM potassium chloride solution (pH 2.0) and siRNA dissolved in nuclease-free water were mixed to obtain a reagent-to-base pair ratio (*R*) of 20. The *R* value was calculated by dividing the nitrogen-to-phosphorus (N/P) ratio by 2. The mixture was incubated at room temperature for 5 min and then mixed with 10× PBS (pH 7.4; Gibco) before injection.

### Animals

All animal experiments were conducted in accordance with the *NIH Guide for the Care and Use of Laboratory Animals* (U.S. Department of Health and Human Services, Public Health Services, National Institute of Health, NIH publication no. 86–23, 1985)^34^, and approved by the Ethical Committee for Animal Experiments at the University of Tokyo. EGFP-overexpressing C57BL/6 mice (C57BL/6-Tg (CAG-EGFP)) were purchased from Japan SLC Inc. C3H/HeN and C3H/HeJ mice were purchased from CLEA Japan. All mice were allowed access to feed and water *ad libitum*.

### DLS and LD analyses

To evaluate the sizes of agglutination, DLS and LD analyses were conducted. DLS measurement was performed on a Malvern Zetasizer Nano ZS equipped with a He-Ne laser operating at 4 mW power and 633 nm wavelength, and a computer-controlled correlator at an accumulation angle of 173°. Measurement was performed at room temperature in a polystyrene or glass cuvette. The data were processed using dispersion technology software version 4.10 to give Z-average particle size and polydispersity index values by cumulant analysis, and particle size distribution by CONTIN analysis^35^,^36^. Laser diffraction (LD) measurement was performed in a Shimadzu Aggregates Sizer equipped with a semiconductor laser operating at 405 nm wavelength. Measurement was carried out at room temperature in a glass cuvette (The light path length is 1 mm). The data were processed using WingSALD bio software version 3.1.1 to give particle size distribution.

DLS samples of TPFE–siRNA complexes were prepared as follows. Mouse serum was prepared just before use. TPFE–siRNA complex (*R* = 20) was prepared using 100 μg of siRNA and was incubated for 5 min at room temperature. The complex was diluted to 200 μL with 10× PBS and water, and then 200 μL of mouse serum was added and the complex was mixed well. The time-dependent change in size was monitored by DLS. The change in size of the TPFE–siRNA complex in the absence of serum was monitored after mixing with PBS.

LD samples of TPFE–siRNA complexes were prepared following the same procedure as that for DLS measurement, and then diluted by 20 times with PBS, due to the limit of concentration. Time-dependent change in size was also monitored by LD.

### Transfection of EGFP-targeted siRNA by TPFE *in vivo*

EGFP-overexpressing mice (age 8–10 weeks) were used for transfection studies. Mice were assigned randomly to either the siGFP + TPFE group or the siNEG + TPFE group. TPFE–siRNA complexes containing 100 μg of siRNA with an *R* value of 20 were injected intravenously. Mice were maintained on feed and water ad libitum. Mice were anesthetized with diethyl ether and sacrificed. Brain, heart, lung, liver, kidney, spleen, and peritoneum tissue specimens were collected in TRIzol reagent and kept at −80°C until use for quantitative real-time RT-PCR.

### Lung histology

To evaluate the accumulation of TPFE–siRNA complexes in the lung, mice were treated with TPFE–siRNA intravenously for 15 min or 3, 12, or 24 h, and the lungs were fixed with 10% neutral buffered formalin via tracheal injection. The fixed lungs were resected and resuspended in 10% neutral buffered formalin. Formalin-fixed tissues were embedded in paraffin. Five-micrometer-thick sections were stained with hematoxylin and eosin (H&E) to stain the nucleus or were left unstained. The accumulation of TPFE–siRNA was determined by measuring the brown-colored areas in 20 fields from unstained lung sections observed at a magnification of 20× on an optical microscope (ECLIPSE 80i; Nikon) equipped with a digital camera (DXM1200F) and Nikon ACT-1 software. The mean area per high-power field (100 × 100 μm^2^) was then calculated using ImageJ software.

To evaluate neutrophil accumulation in the lung, TPFE–siTLR4 complex was injected intravenously into C3H/HeN and C3H/HeJ mice. The C3H/HeN mice were assigned randomly 4 groups; siTLR4 + LPS, siTLR4 + saline, siGFP + LPS or siGFP + saline groups. The C3H/HeJ mice were also divided 2 groups randomly to either the siTLR4 + LPS or the siTLR4 + saline groups. Twenty-four hours after the TPFE–siRNA injection, the mice were treated intratracheally with LPS from *Escherichia coli* serotype O110:B4 (Sigma Aldrich) at a dose of 10 mg/kg or with saline. Six hours later, the mice were anesthetized and sacrificed. The right main bronchus were ligated, and the right lungs were collected, snap-frozen in liquid nitrogen and stored at −80°C until use for determination of MPO and RNAi activities. The left lungs were fixed with 10% neutral buffered formalin via tracheal injection, and the formalin-fixed right lungs were resected and resuspended in 10% neutral buffered formalin. The formalin-fixed tissues were embedded in paraffin. Three-micrometer-thick sections were stained with Giemsa stain and the neutrophils were counted. The sections were analyzed by optical microscopy (ECLIPSE 80i; Nikon). Neutrophils were counted by observing 20 fields at a magnification of 40× in a blinded fashion. The mean number of neutrophils per high-power field was then calculated.

### RNA extraction and quantitative real-time RT-PCR for RNAi activity

Total RNA was extracted using TRIzol reagent. To obtain cDNA of the transcripts, the reverse transcriptase reaction was performed with 1 μg of total RNA (High Capacity cDNA Reverse Transcription Kit, Applied Biosystems). The quantitative real-time RT-PCR was performed with the synthesized cDNA and primers sets for GFP (forward, 5′-GACGTAAACGGCCACAAGTT-3′; reverse, 5′-GGTCTTGTAGTTGCCGTCGT-3′); for TLR4 (forward, 5′-TTTATTCAGAGCCGTTGGTG-3′; reverse, 5′-CAGAGGATTGTCCTCCCATT-3′); and for β-actin as an internal control (forward, 5′-CCACAGCTGAGAGGGAAATC-3′; reverse, 5′-TCTCCAGGGAGGAAGAGGAT-3′) using Power SYBR Green in an ABI7000 Sequence Detection System or Fast Power SYBR Green in a ViiA7 Real-Time PCR System (all from Applied Biosystems). The PCR products were analyzed using Sequence Detection software (version 1.2.3) or ViiA7 software (Applied Biosystems).

### Myeloperoxidase (MPO) activity

MPO activity was measured using a myeloperoxidase fluorometric detection kit (Enzo Life Science) and an fMax fluorescence microplate reader (Molecular Devices) with fMax-pro software according to the manufacturers' protocols. Briefly, lung tissue specimens were homogenized with MicroSmash (TOMY) in 1 mL of assay buffer containing hexadecyltrimethylammonium bromide. The protein concentration in the tissue homogenates was measured using the Micro BCA Protein Assay (Pierce), and 2.5 μg of the lysate was placed in each assay well. All samples were assayed twice in duplicate. The data are presented as units of MPO enzymatic activity per 100 μg of tissue protein.

### Statistical analysis

Differences between the experimental groups were detected using Student's *t* test. Values are expressed as means ± SEM; *P* < 0.05 was considered significant.

## Author Contributions

K.M., E. Noiri, and E. Nakamura designed the research; K.M. and K.O. performed the research; all authors analyzed the data; and K.M., K.D., K.H., E. Noiri, and E. Nakamura wrote the paper.

## Supplementary Material

Supplementary Information Supplementary Information
