# Supplementary material for: siRNA delivery targeting to the lung via agglutination-induced accumulation and clearance of cationic tetraamino fullerene
Source: Sci Rep. 2014 May 12;4:4916. doi: 10.1038/srep04916 (PMC4017229; doi:10.1038/srep04916)
Supplement: Supplementary Information [file srep04916-s1.pdf]

Supplementary Information

siRNA delivery targeting to the lung *via* agglutination-induced accumulation and clearance of cationic tetraamino fullerene

Kosuke Minami,* Koji Okamoto,§ Kent Doi,§ Koji Harano,* Eisei Noiri,§ Eiichi Nakamura*

* *Department of Chemistry, The University of Tokyo, 7-3-1 Hongo, Bunkyo-ku, Tokyo 113-0033, Japan*

§ *Department of Hemodialysis and Apheresis, University Hospital, The University of Tokyo, 7-3-1 Hongo, Bunkyo-ku, Tokyo 113-8655, Japan*

Supplementary Figure

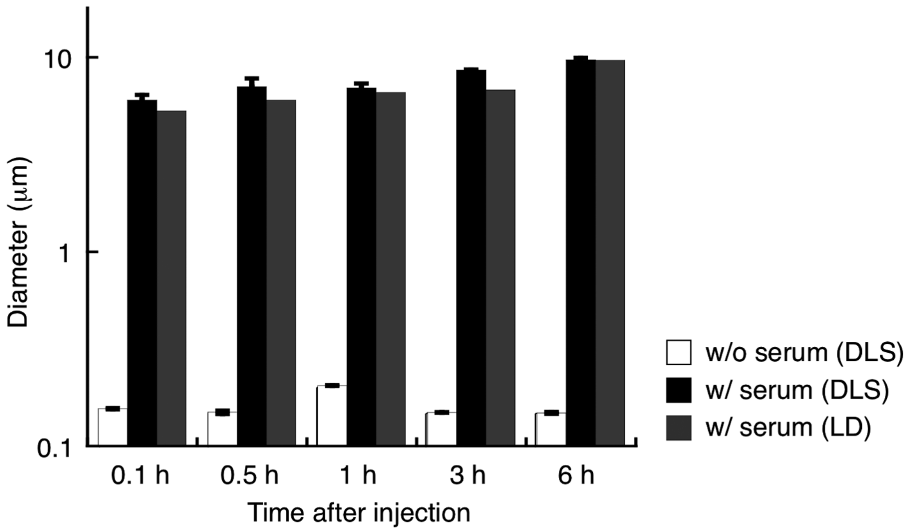

Fig. S1. Agglutination of TPFE-siRNA complex in the serum. Size of TPFE-siRNA complexes was determined by DLS (black and white bars, $N = 3$) and LD (gray bars). Black and gray bars indicate TPFE-siRNA complexes mixed with serum, and white bars indicate the TPFE-siRNA complexes without serum. Error bars indicate ±SD.
